# Increased synovial and entheseal fibroblast activation detected by 68Ga-FAPI-PET/CT is associated with the development of psoriatic arthritis in psoriasis patients with arthralgia

**DOI:** 10.1136/rmdopen-2025-006567

**Published:** 2026-03-23

**Authors:** Giulia Corte, Armin Atzinger, Rita Noversa de Sousa, Melek Yalcin Mutlu, Alp Temiz, Sara Bayat, Maria Gabriella Raimondo, Andreas Ramming, Michael Sticherling, Christian Schmidkonz, Torsten Kuwert, Georg Schett, Koray Tascilar, Filippo Fagni

**Affiliations:** 1 Department of Internal Medicine 3, Friedrich-Alexander University (FAU) Erlangen-Nürnberg and Universitätsklinikum Erlangen, Erlangen, Germany; 2 Deutsches Zentrum Immuntherapie (DZI), Friedrich-Alexander University (FAU) Erlangen-Nürnberg and Universitätsklinikum Erlangen, Erlangen, Germany; 3 Department of Nuclear Medicine, Friedrich-Alexander University (FAU) Erlangen-Nürnberg and Universitätsklinikum Erlangen, Erlangen, Germany; 4 Serviço de Medicina Interna, Hospital Pedro Hispano, Unidade Local de Saúde de Matosinhos, Matosinhos, Portugal; 5 Department of Dermatology, Friedrich-Alexander University (FAU) Erlangen-Nürnberg and Universitätsklinikum Erlangen, Erlangen, Germany; 6 Institute for Medical Engineering, Technical University of Applied Sciences Amberg– Weiden, Weiden, Germany

**Keywords:** Psoriatic Arthritis, Psoriasis, Fibroblasts, Biomarkers, Risk Factors

## Abstract

**Objectives:**

To assess the clinical and imaging characteristics associated with fibroblast activation detected by ^68^Gallium-labelled fibroblast activation protein inhibitor positron emission tomography/CT (^68^Ga-FAPI-PET/CT) in patients with psoriasis and whether ^68^Ga-FAPI uptake correlates with the risk of progression to psoriatic arthritis (PsA).

**Methods:**

Psoriasis patients with arthralgia underwent ^68^Ga-FAPI-PET/CT and were followed up prospectively. ^68^Ga-FAPI uptake was assessed at 71 articular sites and patients with ≥1 joint with ^68^Ga-FAPI uptake and PET/CT Joint Index≥2 were considered FAPI positive. The associations between FAPI uptake and clinical and ultrasound (US) findings were investigated. Survival analyses were conducted to assess the association between ^68^Ga-FAPI uptake and progression to PsA.

**Results:**

45 patients with psoriasis were enrolled, 37 of whom (82%) were FAPI positive. FAPI-positive psoriasis patients had significantly higher body mass index (BMI) (p=0.036) and Disease Activity Score 28-C reactive protein (p=0.033) compared with FAPI-negative patients. ^68^Ga-FAPI uptake was most frequent in large joints and mechanically stressed sites and was more likely in the presence of low-grade synovial hyperplasia (OR: 1.77, 95% CI 1.08 to 2.89), entheseal Power Doppler (OR: 3.80, 95% CI 1.66 to 8.72) and concomitant osteoarthritis (OA). FAPI-positive patients showed a higher risk of progression to PsA compared with FAPI-negative patients (HR 7.1, 95% CI 0.9 to 53.6) (log-rank p=0.028). Only 1/8 patients with psoriasis (12.5%) without ^68^Ga-FAPI uptake developed PsA, as opposed to 18/37 (49%) of FAPI-positive patients.

**Conclusions:**

In psoriasis patients with arthralgia, ^68^Ga-FAPI uptake, indicating fibroblast activation, is associated with higher BMI, more pain, subclinical US changes and concomitant OA. Pathological ^68^Ga-FAPI uptake at articular sites was indicative of higher risk of progression to PsA in our cohort, suggesting fibroblast activation as a crucial step to develop PsA.

**WHAT IS ALREADY KNOWN ON THIS TOPIC**

- Positron emission tomography (PET) tracers based on ^68^Ga-labelled fibroblast activation protein inhibitor (^68^Ga-FAPI) allow us to detect fibroblast activation in humans in vivo. ^68^Ga-FAPI-PET has allowed us to detect stromal tissue activation in various inflammatory rheumatic diseases.
- Psoriasis patients with arthralgia have an increased risk of developing psoriatic arthritis (PsA); however, the pathophysiological events that trigger the onset of PsA are yet incompletely understood.

**WHAT THIS STUDY ADDS**

- In psoriasis patients with arthralgia, ^68^Ga-FAPI-04 uptake was frequently observed at mechanically stressed sites and significantly correlated with higher body mass index, suggesting that biomechanical stress could trigger abnormal fibroblast activation in the joints.
- Psoriasis patients with arthralgia who exhibit increased ^68^Ga-FAPI-04 uptake at articular sites showed a higher risk for developing PsA. Notably, the absence of relevant ^68^Ga-FAPI-04 uptake was highly predictive of non-progression in our cohort.

**HOW THIS STUDY MIGHT AFFECT RESEARCH, PRACTICE OR POLICY**

- This study suggests that ^68^Ga-FAPI-04-PET/CT could be useful for the risk stratification of patients with psoriasis progressing to PsA, enabling the identification of patients who can benefit from closer monitoring and, potentially, early intervention.

## Introduction

 Psoriatic arthritis (PsA) is a chronic and progressive inflammatory joint disease that affects up to 30% of patients with psoriasis and can lead to significant joint damage and functional impairment if not recognised early.^1^ Diagnosing PsA is often challenging due to the heterogeneity of its manifestations and due to its frequently insidious onset. At this early stage, patients with psoriasis experience non-specific musculoskeletal symptoms such as arthralgia and stiffness and show subclinical inflammatory changes on imaging, but still have no clinical signs of arthritis.^2^ This subclinical phase theoretically represents a window of opportunity during which patients with psoriasis could benefit from preventive intervention.^3 4^ While it is known that a phase of discrete inflammation in joints and entheses precedes the onset of PsA, little is known about the molecular processes in the joints that are associated with its onset, as conventional imaging methods, like ultrasound (US) and MRI, are detecting and quantifying inflammation rather than molecular changes in the target tissues.

Dependent on the tracer used, positron emission tomography (PET) allows us to detect molecules and molecular processes in target tissues.^5^ ^68^Gallium-labelled fibroblast activation protein inhibitor (^68^Ga-FAPI) allows us to detect stromal tissue responses in humans in vivo, as the tracer exclusively binds to activated fibroblasts. Hence, stromal tissue activation in association with malignant, fibrotic and chronic inflammatory phenomena can be detected by ^68^Ga-FAPI PET, which, in conjunction with CT, allows a precise anatomical localisation.^6^ The feasibility to use ^68^Ga-FAPI PET/CT to detect stromal tissue activation in rheumatic diseases has been demonstrated previously in rheumatoid arthritis (RA) and early RA, IgG4-related disease, systemic sclerosis and myositis, enabling whole-body ‘staging’ of joint involvement in a single session.^6^,^10^

In a pilot study, we showed that ^68^Ga-FAPI-PET/CT can detect fibroblast activation at articular sites of patients with psoriasis and arthralgia. In these patients, the intensity of ^68^Ga-FAPI uptake correlated with the presence of tenderness at clinical examination.^11 12^ With this exploratory prospective cohort study, we aim to elucidate what clinical and disease-related characteristics influence ^68^Ga-FAPI uptake at articular sites in psoriasis patients with arthralgia, and whether ^68^Ga-FAPI uptake predicts the development of PsA.

## Methods

### Patient recruitment

We assessed the eligibility of adult patients with a diagnosis of psoriasis confirmed through histology and/or by a dermatologist who were referred to the outpatient clinic of the Department of Rheumatology and Immunology at the University Hospital of Erlangen (Germany), between October 2022 and June 2024, because of new onset of musculoskeletal symptoms to rule out PsA. All patients underwent clinical examination, laboratory testing and, when deemed necessary, an US examination by an experienced rheumatologist (GC or FF) in order to exclude the presence of PsA and assess subclinical US changes. Only patients with arthralgia persisting for at least 3 months and no clinical signs of inflammatory joint involvement who had never satisfied the CASPAR criteria,^13^ based on their medical history and at the time of the visit, were included.

Patients with positive rheumatoid factor or anticitrullinated protein antibodies were excluded. Patients with overlapping diagnoses potentially related to the reported musculoskeletal symptoms, including immune-mediated or metabolic inflammatory diseases (such as RA, inflammatory connective tissue diseases, vasculitis, systemic autoinflammatory diseases and crystal arthropathies), were excluded, as were patients with a diagnosis of fibromyalgia or musculoskeletal complaints localised exclusively in joints with documented osteoarthritis (OA). Patients who were pregnant or planning to become pregnant were excluded.

The use of conventional, biologic and targeted synthetic disease-modifying antirheumatic drugs (cs/b/tsDMARDs) was allowed as needed due to skin disease activity, provided the dose remained stable in the 6 months preceding study entry.

Eligible patients were included in the study after providing written informed consent and were referred for further imaging with ^68^Ga-FAPI-PET/CT.

After ^68^Ga-FAPI-PET/CT acquisition, clinical examinations were conducted every 3 months or on demand in case of worsening of symptoms or possible joint swelling. The diagnosis of PsA was based on fulfilment of the Classification Criteria for PsA (CASPAR), considering joint involvement as the presence of clinical synovitis, axial involvement as confirmed by MRI and entheseal involvement as US-confirmed inflammation at clinically tender entheses. To be included in the final analyses, patients had to provide a minimum follow-up of 6 months or develop PsA after the PET/CT scan.

Clinicians involved in the study were not blinded to the imaging results at follow-up. All participants were enrolled in the psoriasis and PsA cohort of the University Clinic of Erlangen (#86_21 Bc), as detailed elsewhere.^14^ An overview of inclusion and exclusion criteria is provided in online supplemental table 1.

### Clinical measurements

Demographic data on age, sex, body mass index (BMI), physical activity, family history of psoriasis, as well as data on disease-related factors, including the presence of nail and scalp involvement, duration of psoriasis and of arthralgia and ongoing therapies, were collected at baseline. Core-set measures of disease activity and patient-reported outcome measures were assessed at each timepoint. Tender joint count (TJC) and swollen joint count were assessed as the affected count among 66/68 respective joints, and entheseal tenderness was measured using Maastricht Ankylosing Spondylitis Enthesitis Score (MASES), Leeds Enthesitis Index (LEI) and Spondyloarthritis Research Consortium of Canada (SPARCC) Enthesitis Index. Axial symptoms were assessed by the Bath Ankylosing Spondylitis Disease Activity Index (BASDAI). Psoriatic skin disease activity was assessed with the Psoriasis Area Severity Index (PASI). Patient-reported pain intensity and Global Health were measured on a 0–100 Visual Analogue Scale (VAS), and the duration of morning stiffness was reported in minutes. Systemic inflammatory activity was assessed by C reactive protein (CRP) levels (mg/L). The Health Assessment Questionnaire was used to assess disability. Data on ongoing systemic therapy for psoriasis were recorded.

### US examination

The US examinations were performed by one of two rheumatologists experienced in musculoskeletal US (GC, 7 years of experience; FF, 4 years of experience), using either a MyLab V.25 or a MyLab Twice device (Esaote Biomedica, Genoa Italy) equipped with a 3–16 MHz and 4–20 MHz linear transducer, respectively. Standardised bilateral US examinations of all the following sites were performed at the baseline examination: wrist, metacarpophalangeal joints, finger flexor tendons, elbow and the enthesis at lateral epicondyle, knee and distal quadriceps tendon enthesis, distal patellar tendon enthesis and Achilles’ tendon enthesis. US examinations were performed according to European Alliance of Associations for Rheumatology scanning guidelines, employing both B-mode and Power Doppler (PD) mode.^15^ We assessed and scored the synovitis in four grades (0–3) and evaluated the presence of inflammatory (PD sign, thickening, hypo-echogenicity) and structural changes (erosion and enthesiophytes/classifications) at entheseal sites, according to the Outcome Measures in Rheumatology (OMERACT) definitions and scoring systems.^16^ Grade 1 synovitis without the PD signal was considered non-specific. The classification of enthesitis was based on the proposed definition of active enthesitis, defined as the presence of either PD Grade 1 plus entheseal thickening and/or hypoechoic areas or PD Grade≥2 independent of other findings.^17^

### ^68^Ga-FAPI-PET/CT examination

^68^Ga-FAPI PET/CT scans were performed with the Biograph Vision 600 system (Siemens Healthineers, Erlangen, Germany) using either ^68^Ga-FAPI-04 or ^68^Ga-FAPI-46 as tracers. ^68^Ga-FAPI-04 was used under the auspices of the §13 (2b) of the German Medicinal Products Act (Arzneimittelgesetz) and synthesised following the guidelines of the Good Manufacturing Practice, as reported previously.^18^ The imaging protocol covered the entire body from the skull to the toes, with an additional bed position to include the hands, each acquired for 3 min (axial field of view per bed: 26.3 cm). The estimated radiation exposure for a 68Ga-FAPI PET/CT scan was 6–8 mSv. Data corrections for random and scattered coincidences, as well as for decay during scanning, were applied. Attenuation correction was performed using the CT component of the multimodal acquisition. Reconstruction and correction processes were conducted using the manufacturer’s software. Subsequent analyses were carried out using Syngo.via software (Siemens Molecular Imaging, Hoffman Estates, Illinois, USA), which facilitated the review of PET, CT and fused imaging data. Visual evaluations were performed by two experienced nuclear medicine physicians (AA and CS) and one radiologist, all blinded to clinical data. Regions of interest (ROIs) were assessed through visual interpretation of coronal, sagittal and transverse slices.

In each patient, we evaluated 34 synovial, 32 entheseal and 5 axial sites for the presence of ^8^Ga-FAPI uptake. The complete list of ROIs examined is provided in the online supplemental materials. Following the methodology described by Luo *et al*,^8^ a circular volume of interest (VOI) was placed around the affected joint to determine the standardised uptake value (SUVmax). In this study, the SUVmax was used, representing the measurement of the most intense pixel within the selected VOI. Additionally, an FAPI-PET/CT Joint Index (PET-JI) was calculated, comparing the intra-articular tracer uptake intensity at each ROI with that of the blood pool and gluteal muscle, which served as reference organs.^8^ Patients were classified as ‘^68^Ga-FAPI-PET/CT positive’ when at least one joint or enthesis exhibited significant radionuclide uptake, defined as equal to or greater than two times the uptake of the gluteal muscle (PET-JI≥2). The presence of bony changes attributable to OA was evaluated by CT at each synovial site. The assessment of CT signs of OA was performed by a rheumatologist (FF) based on established radiological criteria, including joint space narrowing, subchondral sclerosis, osteophyte formation and subchondral cysts.

### Statistical methods

Descriptive data were analysed using means and SDs or medians and IQRs for continuous variables and count data, and percentages were used for categorical variables. Between-group comparisons based on any versus no uptake were conducted using the Wilcoxon’s rank-sum test or Fisher’s exact test as appropriate. The association between sonographic findings and FAPI uptake was analysed at the level of each corresponding anatomical site where FAPI and sonography data were mutually available. A separate generalised additive model with logit link function was fit for each type of sonographic finding and separately for joints and entheseal sites and separately for the joint index and SUV, where the dependent variable was a binary indicator for the presence of specific sonographic findings such as synovial hypertrophy and erosions and the independent variable was an FAPI uptake quantity, that is, joint index or SUV. We used a random-effects smoother to account for within-person correlation and calculated ORs for observing a given sonographic finding associated with increasing FAPI uptake.

The risk of developing PsA over time was analysed using survival methods. We generated Kaplan-Meier plots depicting PsA-free survival by baseline FAPI uptake status and the presence of sonographic findings. Cox regression was used to calculate the HRs of developing PsA by overall patient-level uptake quantities as well as subsets of these quantities, such as uptake at peripheral entheses, axial skeleton or joints separately. Due to possible confounding by weight, we also conducted analyses adjusted for BMI, but these showed virtually the same findings and were therefore omitted.

Analyses were conducted on complete datasets without missing data imputation. Two-sided p values less than 0.05 and 95% CIs excluding a null effect were considered statistically significant. We used the open-source R software (V.4.5.1, R Foundation for Statistical Computing, Vienna, Austria) running under the integrated development environment RStudio (V.2025.05.01, Posit Software, Boston Massachusetts, USA). Key packages used for the analyses were survival (V.3.8), survminer (V.0.5) and mgcv (V.1.9).

## Results

### Patient characteristics

**Figure 1:**
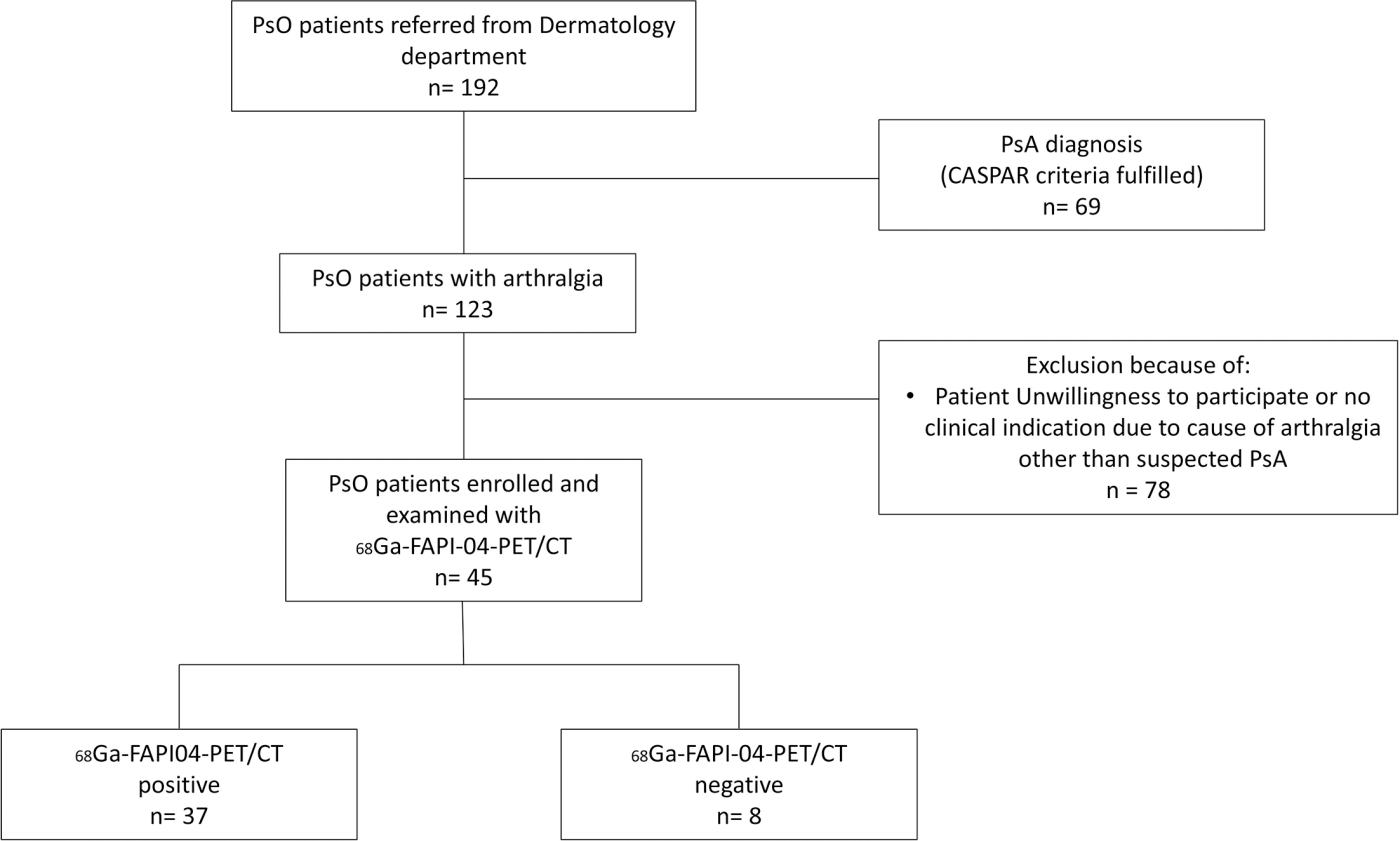
Flow chart of patient selection and inclusion into the study cohort. CASPAR, Classification Criteria for PsA; ^68^Ga-FAPI04-PET/CT, ^68^Gallium-labelled fibroblast activation protein inhibitor-04 positron emission tomography/CT; PsA, psoriatic arthritis; PsO, psoriasis.

192 patients with psoriasis were assessed to exclude the presence of PsA. Of them, 69/192 (35.9%) were excluded as they already had signs and symptoms of PsA or fulfilled the CASPAR criteria on first presentation. A further 78/192 patients (40.6%) were excluded due to the presence of overlapping musculoskeletal conditions or unwillingness to participate in the study. 45 out of 192 patients (23.4%) were included in the study and underwent ^68^Ga-FAPI-PET/CT examination (figure 1).

Participants had a mean (SD) age of 50 (12) years and 19 (42.2%) were women. Most had longstanding psoriasis with a median (IQR) disease duration of 16 (6–33) years and onset of arthralgia of a median duration of 3 (2–7) years. The mean BMI was 29.1 (6.6) kg/m^2^ in the upper overweight range. The majority of patients reported a sedentary lifestyle (n=24, 53.3%), while the remaining 19 exercised occasionally or regularly. The tender joint and enthesis counts at baseline were low on average, with a TJC of 1.0 (0.0–4.0) and Disease Activity Score 28 (DAS28) of 2.65 (1.3). Most patients had no clinical entheseal tenderness based on the proportion of patients with 0 score on the LEI (26, 57.8%), MASES (n=34, 75.6%) and SPARCC (n=23, 51.1%) indices. The median SPARCC was 0 (IQR: 0–2). Five joints in two patients (one knee and four distal interphalangeal joints) were assessed as swollen due to OA, but no US evidence of synovitis or X-ray changes suggestive of PsA was found. Low-back pain was relatively prevalent throughout the cohort, and the mean BASDAI was 3.4 (2.3). Skin disease activity was variable throughout the cohort, with a mean PASI of 2.2 (2.6). On average, patients reported moderate levels of pain and perceived activity with a mean VAS pain of 40 (25) mm and VAS Global Health of 39 (25) mm. Genetic testing was available for 23/45 patients (51.1%), of whom 2 tested positive for the human leukocyte antigen (HLA)-B27 and 5 for interleukin 23-receptor polymorphisms (IL23-R). At presentation, just one patient was using methotrexate for psoriasis treatment, while 15/45 (33.3%) were under therapy with b/tsDMARDs. Detailed clinical characteristics are summarised in table 1.

**Table 1:** Demographics and disease-related characteristics

	Overall n=45	Positive n=37	Negative n=8	P value
Age (years), mean (SD)	50 (12)	52 (12)	47 (10)	0.8
Male/female (N)	26/19	21/16	5/3	>0.9
BMI, mean (SD)	29.1 (6.6)	29.9 (6.8)	25.5 (3.9)	**0.036**
Physical activity, N (%)				
Sedentary or <once weekly	24 (57)	20 (57)	4 (57)	>0.9
Once weekly to daily	10 (21)	8 (20)	2 (29)	
Daily	9 (21)	8 (23)	1 (14)	
Family history of psoriasis	21 (47)	16 (43)	5 (6)	0.7
Duration of psoriasis (years), mean (SD)	20 (17)	18 (17)	27 (17)	0.2
Duration of arthralgia (years), mean (SD)	7 (11)	8 (12)	4 (6)	0.4
VAS pain (0–100), mean (SD)	40 (25)	43 (25)	26 (21)	0.09
VAS Global Health (0–100), mean (SD)	38 (26)	41 (23)	29 (16)	0.22
TJC (68), median (IQR)	1.0 (0.0, 4.0)	1.0 (0.0, 4.0)	0.5 (0.0, 2.0)	0.6
SJC (66), median (IQR)	0.0 (0.0, 0.0)	0.0 (0.0, 0.0)	0.0 (0.0, 0.0)	–
LEI>0, N (%)	18 (40)	16 (43)	2 (25)	0.4
MASES>0, N (%)	10 (22)	8 (22)	2 (25)	>0.9
SPARCC>0, N (%)	21 (47)	18 (49)	3 (37)	0.7
BASDAI, mean (SD)	3.4 (2.3)	3.4 (2.4)	3.6 (2.2)	0.7
DAS28-CRP, mean (SD)	2.65 (1.3)	2.82 (1.2)	1.75 (1.5)	**0.033**
CRP>5 mg/L, N (%)	16 (36)	15 (41)	1 (13)	
mg/L, mean (SD)	8.0 (6.5)	8.9 (7.5)	5.6 (1.6)	0.2
Psoriasis patterns, N (%)				
Nail involvement	13 (31)	9 (26)	4 (50)	0.2
Scalp involvement	24 (56)	20 (57)	4 (50)	>0.9
PASI, mean (SD)	2.2 (2.6)	2.5 (2.8)	1.2 (1.2)	0.2
Ongoing therapy, N (%)				
No systemic therapy	2 (62)	24 (65)	4 (50)	0.7
cDMARD	1 (2)	1 (3)	0 (0)	>0.9
bDMARD	14 (32)	11 (30)	3 (38)	0.7
tsDMARD	2 (4)	1 (3)	1 (13)	0.3

Percentages were calculated using the total available data. Significant values are highlighted in bold.

BASDAI, Bath Ankylosing Spondylitis Disease Activity Index; BMI, body mass index; c/b/ts DMARD, conventional, biological and targeted synthetic disease-modifying antirheumatic drugs; CRP, C reactive protein; DAS28, Disease Activity Score 28; LEI, Leeds Enthesitis Index; MASES, Maastricht Ankylosing Spondylitis Enthesitis Score; PASI, Psoriasis Area Severity Index; SJC, swollen joint count; SPARCC, Spondyloarthritis Research Consortium of Canada; TJC68, count of 68 tender joints; VAS, Visual Analogue Scale.

### ^68^Ga-FAPI-PET/CT findings

**Figure 2:**
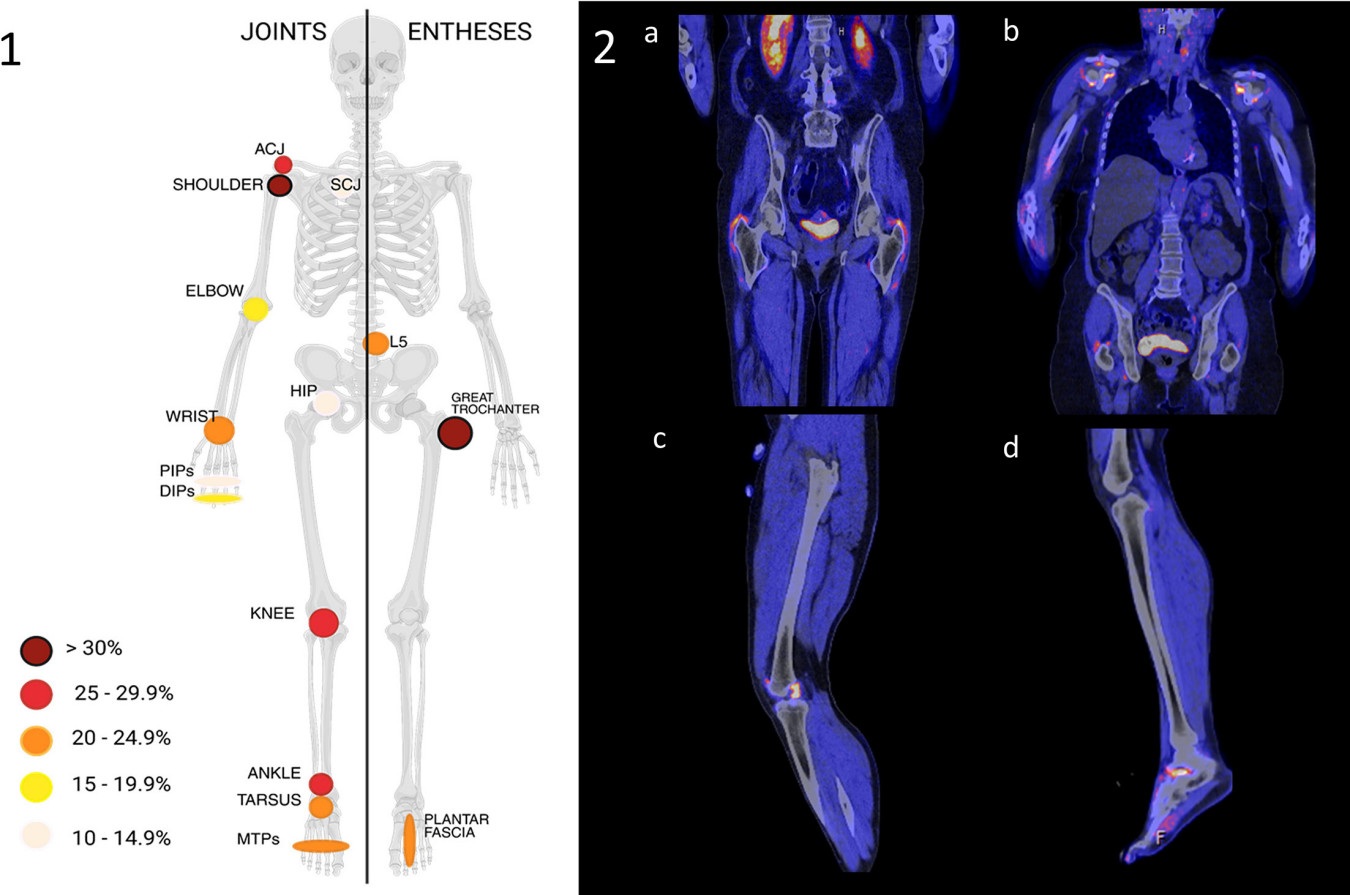
Anatomical distribution of ^68^Gallium-labelled fibroblast activation protein inhibitor (^68^Ga-FAPI) uptake. (1) Graphical representation of the 15 sites that most commonly showed increased fibroblast activation in patients with psoriasis. The colour scale reflects the site-specific frequency of ^68^Ga-FAPI uptake expressed as a percentage on the total of all patients: dark red: >30%; light red: 25–29.9%; orange: 20–24.9%; yellow: 15–19.9%; white: 10–14.9%. (2) PET/CT images of sites that most commonly show increased ^68^Ga-FAPI uptake: great trochanter (a), shoulder (b), knee (c) and ankle (d). ACJ, acromioclavicular joint; DIPs, distal interphalangeal joints; MTPs, metatarsophalangeal joints; PET/CT, positron emission tomography /computed tomography; PIPs, proximal interphalangeal joints; SCJ, sternoclavicular joint.

A total of 252/3195 (7.9%) sites displayed increased radiotracer uptake. The majority (37/45) of patients showed uptake of FAPI in at least one site, while 8 out of 45 patients had no relevant signs of fibroblast activation at FAPI-PET/CT. The mean total SUVmax burden, that is, the sum of all SUVs in all examined sites per patient, was 22 (24). The most commonly affected sites were the lower extremities and weight-bearing joints. The most frequently involved entheses were at the greater trochanter (n=15/45), dorsal process of L5 vertebra (n=10/45) and plantar fascia (n=9/45). As for joints, ^68^Ga-FAPI uptake was most frequent at the shoulder (n=14/45), ankles (n=13/45), knees (n=13/45) and acromioclavicular joint (n=12/45). A detailed overview of ^68^Ga-FAPI uptake at synovial, entheseal and axial sites is shown in figure 2 and online supplemental table 2.

CT signs of OA were present in 109 of 3195 total sites (3.4%) and in 72 out of 282 joints with increased ^68^Ga-FAPI uptake (25.5%). After excluding ^68^Ga-FAPI uptake related to OA, the proportion of sites showing radiotracer uptake in lower and upper extremities was more similar (online supplemental table 2).

### ^68^Ga-FAPI-PET/CT uptake and clinical findings

Psoriasis patients with evidence of fibroblast activation at sites in the ^68^Ga-FAPI-PET/CT differed significantly in some clinical aspects from patients who had no ^68^Ga-FAPI uptake. FAPI-positive patients had significantly higher BMI (p=0.036), higher levels of pain (p=0.085) and higher DAS28 (p=0.033). Demographic factors, including age, sex distribution and physical activity levels, were comparable between the groups. Other disease-related factors such as TJC, LEI, MASES, SPARCC type of psoriasis involvement, PASI and CRP, as well as ongoing therapies, did not differ between the groups (table 1). However, each one-point increase in the PET-JI was associated with joint or entheseal tenderness with an OR of 1.71 (1.28–2.29, p<0.001), as well as one-point increase in SUV with an OR of 1.40 (95% CI 1.19 to 1.65, p<0.001). In the subgroup of patients for whom a genetic examination was available, there was no difference in the frequency of HLA-B27 or IL23-R polymorphism.

### US characteristics and correlation with ^68^Ga-FAPI uptake

We acquired 385 greyscale and PD US scans for synovial joints, 124 greyscale and PD scans for finger flexor tendons and 256 greyscale and PD scans of the entheses from 23 patients. Grade 1 synovial hypertrophy was present in 24/385 sites (6.2). A grade 1 PD signal was detected at 8/385 sites (2.1%). Greyscale signs of tenosynovitis were found in 3/124 extensor tendons (2.4%) without the PD signal. Regarding entheses, hypo-echogenicity and calcifications/enthesophytes were the most prevalent findings, found at 32/256 (12.5%) and 27/256 (10.5%) sites, respectively. Detailed information on the prevalence and distribution of US changes is shown in online supplemental table 3.

**Figure 3:**
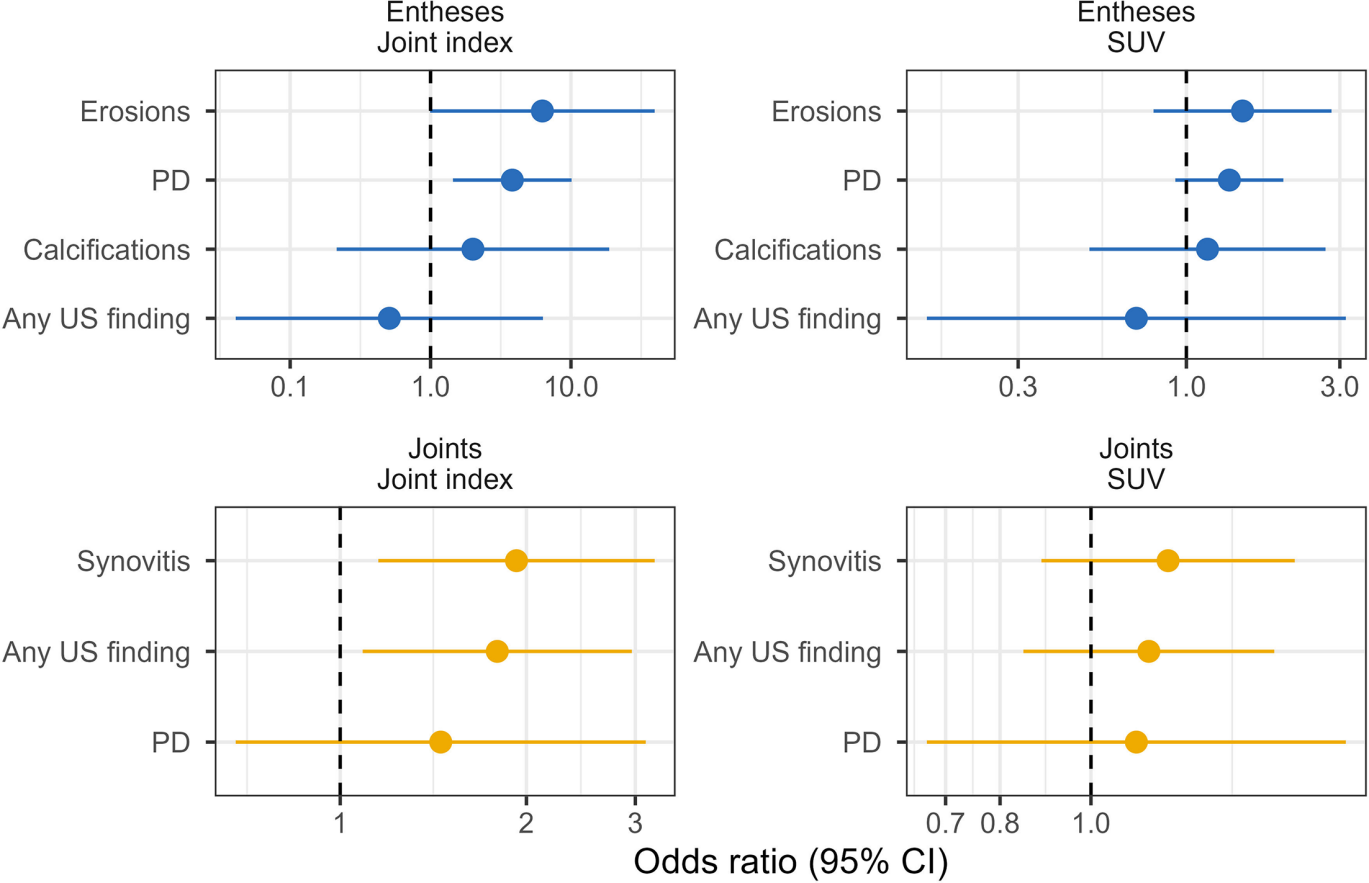
Association between SUV/joint index and individual sonographic findings. The forest plot shows the ORs and 95% CIs for sonographic findings per 1 SD increase in ^68^Gallium-labelled fibroblast activation protein inhibitor uptake measured by joint index (left) and total SUV (right) at the same site in synovial joints and entheses. PD, Power Doppler; SUV, standardised uptake value; US, ultrasound.

Uptake of ^68^Ga-FAPI at synovial and entheseal sites was associated with specific US abnormalities. In synovial joints, each one-point increase in the joint index was linked to synovial hypertrophy on US (OR 1.77; 95% CI 1.08 to 2.89). At entheseal sites, the presence of a power PD signal was associated with an OR of 3.80 (95% CI 1.66 to 8.72) (figure 3). No further US findings were otherwise significantly associated with ^68^Ga-FAPI uptake. Detailed information on the association of the FAPI signal with sonographic findings is reported in online supplemental table 3.

### ^68^Ga-FAPI-PET/CT uptake and PsA development

The total follow-up time of our cohort amounted to 552 patient-months corresponding to a median of 33 (IQR 14–62) weeks. 20 out of 45 patients developed clinical PsA. The median time to diagnosis was 10 (4–48) weeks. The diagnosis of PsA was enabled by clinical evidence of synovitis due to joint swelling and tenderness or enthesitis, the latter always confirmed by US. Among the 20 patients who developed PsA, 19 showed FAPI uptake in any joint examined, whereas 1 did not.

**Figure 4:**
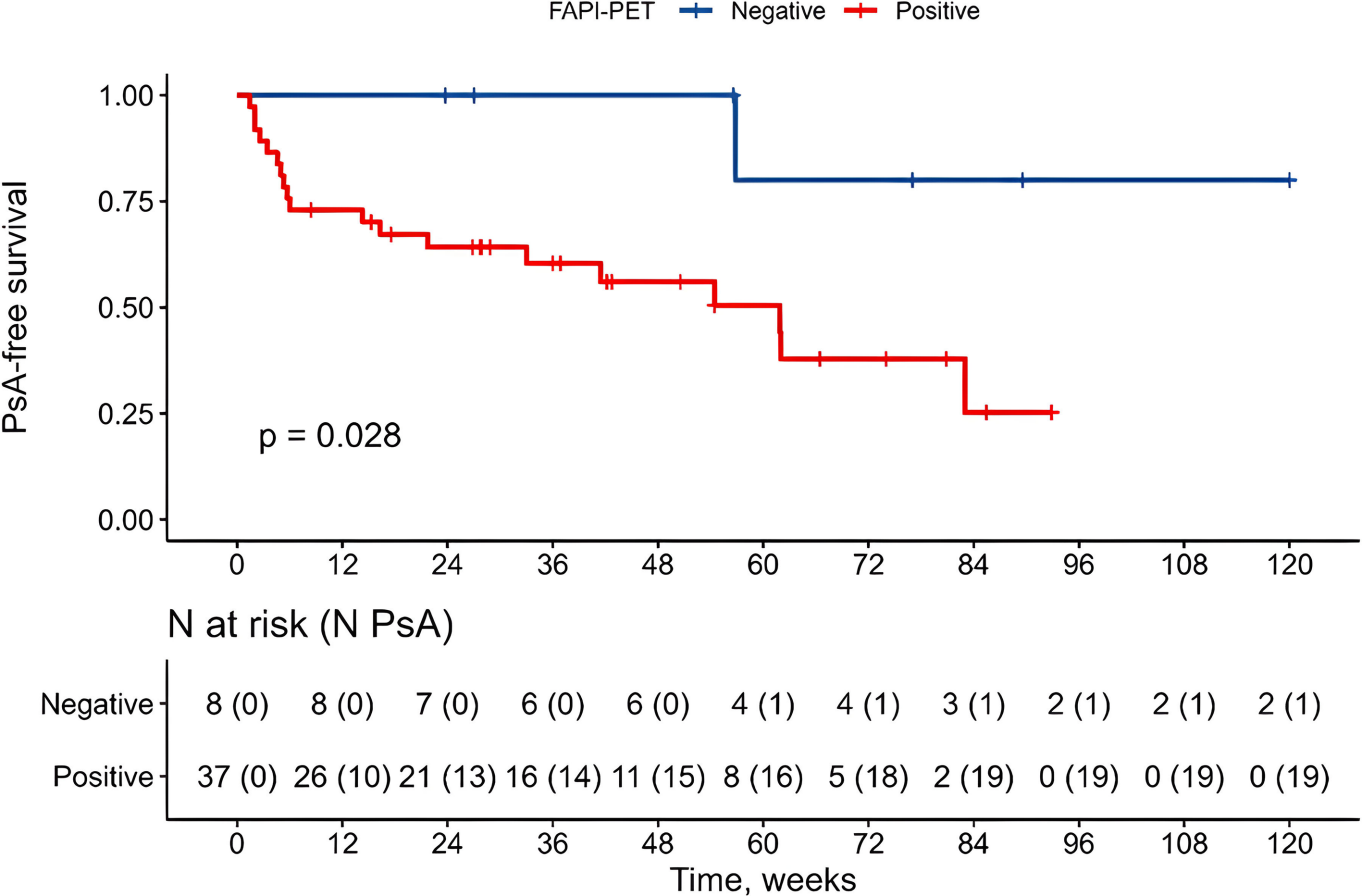
Kaplan-Meier survival estimates for psoriatic arthritis (PsA) development by fibroblast activation protein inhibitor positron emission tomography (FAPI-PET) positivity. The red line represents patients with relevant ^68^Gallium-labelled FAPI uptake, categorised as FAPI-PET positive, while the blue line represents FAPI-PET-negative patients. The y-axis shows the probability of PsA-free survival. The x-axis represents follow-up time in months. P value was calculated with a log-rank test.

Survival analyses were performed comparing two groups of patients based on the presence of relevant ^68^Ga-FAPI uptake at sites. Patients with FAPI uptake in at least one site (ie, FAPI positive) developed PsA in 19/37 (51.4%) of cases. Of the remaining eight FAPI-negative patients, only one (12.5%) developed PsA. By the end of follow-up, the cumulative probability of PsA-free survival was 25% (95% CI 6% to 66%) among patients who showed uptake compared with 80% (95% CI 52% to 100%) among patients who showed no uptake (log-rank p=0.028) (figure 4). This corresponded to an HR of PsA development of 7.1 (95% CI 0.9 to 53.6) with FAPI uptake.

To assess the predictive value of PET/CT compared with articular US, we repeated the survival analyses based on the presence or absence of US abnormalities at joints or entheses. Since further imaging was indication-based, this analysis included a subset of 23 patients who had undergone a comprehensive bilateral US assessment covering at least 40 synovial and entheseal sites. In our cohort, US showed no significant predictive value for the development of PsA among those undergoing US evaluation. We observed 1/19 progression events among patients with US abnormalities, compared with 2/4 progression events in patients with normal US findings (p=0.13; online supplemental figure 1).

**Figure 5:**
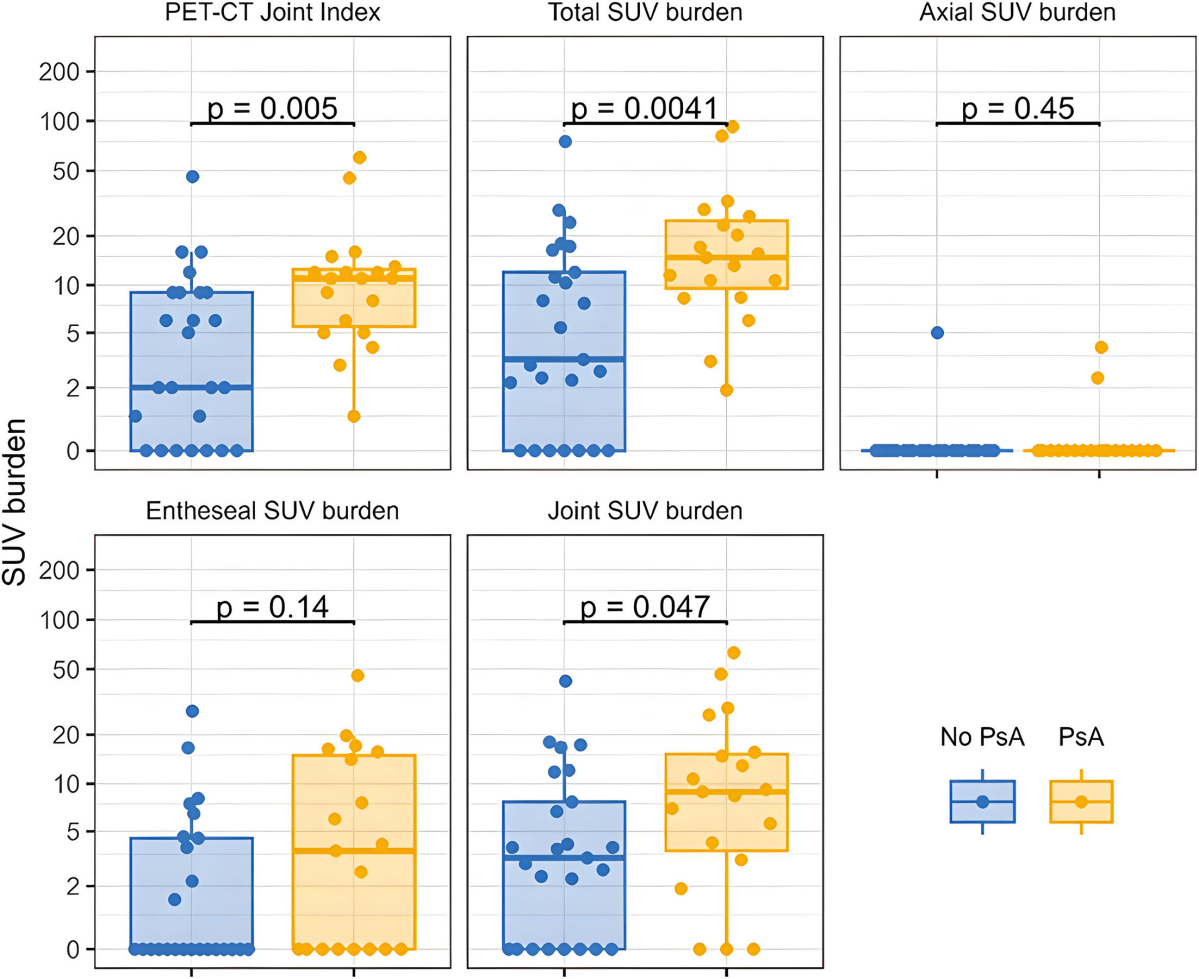
Patient-level total standardised uptake value (SUV) and positron emission tomography (PET)-CT Joint Index burden by psoriatic arthritis (PsA) development. The box plot shows the individual data points, median values and IQRs of the total joint index and of the total SUV values at synovial, entheseal and axial sites per patient. Only sites without osteoarthritis were considered. Patients who did not develop PsA are represented in blue, and progressors to PsA are shown in orange. Comparisons were made with the Wilcoxon rank-sum test. The y-axis is pseudo-log transformed to improve visibility.

When considering all joints including those affected by OA, patients who developed PsA had an overall higher total joint/entheseal SUV burden and slightly greater overall PET-JI compared with patients with psoriasis without arthritis, but differences were not statistically significant (online supplemental table 4). After excluding ^68^Ga-FAPI uptake due to OA, differences between the two groups became significant. We observed a mean total SUV burden of 22 (SD: 24) in the PsA progressor group as opposed to 10 (SD: 16) in non-progressors (p=0.004). PsA progressors had a significantly higher SUV burden at synovial joints (14 vs 6, p=0.047) and a numerically higher SUV burden at the entheses (8.0 vs 3.3, p=0.14) than non-progressors. Uptake at axial sites did not differ between groups. Detailed information on SUV burden for each group is visualised graphically in figure 5. In general, each point increase of the total SUV burden was linked to an HR of 1.3 (95% CI 1.1 to 1.5) of developing PsA, and each point increase of the PET-JI had an HR of 1.4 (95% CI 1.0 to 1.8). Specifically, the presence of ^68^Ga-FAPI uptake at synovial joints without OA was associated with an HR of 1.4 (95% CI 1.0 to 1.8) of developing PsA, while uptake at entheseal sites corresponded to an HR of 1.7 (95% CI 1.1 to 2.7).

## Discussion

Tissue-resident fibroblasts play a central role in the establishment of inflammatory arthritis by secreting proinflammatory cytokines and chemokines and thus promote immune cell migration in synovial and entheseal tissues.^19 20^ In this exploratory prospective cohort study, we showed that ^68^Ga-FAPI-PET/CT reveals increased fibroblast activation at sites before the onset of arthritis, and that this increased fibroblast activity was predictive of PsA development.

Confirming previous preliminary observations from our group,^11 12^ we observed that large joints and weight-bearing sites were most frequently affected by ^68^Ga-FAPI uptake and that FAPI-positive patients presented a significantly higher BMI compared with patients with a negative ^68^Ga-FAPI PET/CT scan. These data raise the hypothesis that biomechanical stress and metabolic factors in psoriatic disease^21 22^ may contribute to abnormal stromal tissue activation and the very early stages of inflammation in PsA, as also postulated by McGonagle *et al*.^23^ This concept also suggests that the influence of metabolic factors in establishing and sustaining PsA could be mediated by alterations in fibroblast activity.^24^ Furthermore, ^68^Ga-FAPI uptake was associated with the presence of subclinical inflammatory US changes. Notably, the detection of ^68^Ga-FAPI uptake in joints and entheses was more frequent compared with the prevalence of US changes at the same sites. Considering that the US assessment was indication-based, a valid comparison is precluded. However, US was performed in a standardised manner, encompassing the same synovial and entheseal sites for all patients. Thus, it is plausible that ^68^Ga-FAPI-PET/CT might capture earlier stromal tissue changes underlying the inflammatory process that are not otherwise visible on US.

During follow-up, patients who displayed ^68^Ga-FAPI uptake demonstrated a higher risk of developing PsA compared with patients with psoriasis who did not show imaging signs of fibroblast activation. Only one of the patients with negative ^68^Ga-FAPI PET/CT developed PsA, highlighting that, in our cohort, ^68^Ga-FAPI-PET/CT provided a high negative predictive value in assessing future PsA development. Increased whole-body SUV burden was significantly associated with PsA development, while restricting the analysis to synovial or entheseal sites alone did not improve discriminatory performance. Taken together, these findings support the concept that fibroblast activation may play a central pathogenetic role in the transition from psoriasis to PsA.

Nonetheless, additional steps are needed to fully understand how and when increased stromal tissue remodelling translates into PsA and what influences fibroblast activation. For instance, we describe that joints affected by OA also have increased ^68^Ga-FAPI uptake. On one side, this did not significantly influence the survival analyses with regard to the likelihood of PsA development by the overall presence or absence of imaging changes. However, the presence of radionuclide uptake due to OA represented a confounding factor in interpreting the correlation between cumulative whole-body ^68^Ga-FAPI uptake and the risk of PsA development. In fact, a significant correlation between cumulative SUV and development of PsA was found only when the uptake in the joints affected by OA was excluded from the analysis, suggesting that fibroblast activation in osteoarthritic joints of patients with psoriasis does not confer in itself an increased risk of progression to PsA. Therefore, the role of OA in inducing fibroblast activation detectable by ^68^Ga-FAPI PET/CT deserves to be assessed separately.

Our study has some limitations. First, our cohort was relatively small and included highly selected patients with psoriasis at high risk of developing PsA (ie, high average pain ratings, high BMI, frequent nail and scalp involvement), providing a selection bias that limits the generalisability of our findings. However, this provided us with a relatively high event rate due to the high baseline risk. The clinical investigators were not blinded to the ^68^Ga-FAPI PET/CT results, as clinical follow-up necessitated a degree of unblinded evaluation to ensure appropriate counselling. To mitigate this bias, we employed standardised protocols for data collection and imaging results were evaluated by an independent assessor with no access to patient data. Hence, we cannot exclude the possibility of confirmation bias. Furthermore, the absence of a group comparison with other musculoskeletal diseases including OA limits the specificity of our findings. Lastly, the evaluation of OA in CT has limited sensitivity for small joints, which could have led to an underestimation of FAPI-positive OA. Radiation exposure and relatively high costs are inherent technical limitations that still need to be overcome.

In conclusion, our study shows that ^68^Ga-FAPI-PET/CT can be used to visualise fibroblast activation at articular sites in vivo and that this resident tissue activation was associated with a higher rate of progression to PsA in our cohort. These data suggest that ^68^Ga-FAPI-PET/CT could have potential for the risk stratification of patients with psoriasis at the highest risk of progression to PsA, pinpointing patients who may benefit from closer rheumatological monitoring and, possibly, from early and/or preventive therapeutical intervention. Future studies should address the suitability of ^68^Ga-FAPI for assessing disease activity and treatment response in PsA and to define the role of other factors influencing ^68^Ga-FAPI uptake, such as body weight, concomitant OA and other overlapping conditions. Lastly, ^68^Ga-FAPI-PET/CT needs to be assessed in other musculoskeletal conditions to better understand the significance of fibroblast activation in arthritis.

## Supplementary material

10.1136/rmdopen-2025-006567 online supplemental material 1

## Data Availability

Data are available upon reasonable request.

## References

[1] Ritchlin CT, Colbert RA, Gladman DD (2017). Psoriatic Arthritis. N Engl J Med.

[2] De Marco G, Zabotti A, Baraliakos X (2023). Characterisation of prodromal and very early psoriatic arthritis: a systematic literature review informing a EULAR taskforce. RMD Open.

[3] Scher JU, Ogdie A, Merola JF (2019). Preventing psoriatic arthritis: focusing on patients with psoriasis at increased risk of transition. Nat Rev Rheumatol.

[4] López-Medina C, McGonagle D, Gossec L (2025). Subclinical psoriatic arthritis and disease interception—where are we in 2024?. Rheumatology (Oxford).

[5] Noversa de Sousa R, Tascilar K, Corte G (2024). Metabolic and molecular imaging in inflammatory arthritis. RMD Open.

[6] Schmidkonz C, Kuwert T, Atzinger A (2022). Fibroblast Activation Protein Inhibitor Imaging in Nonmalignant Diseases: A New Perspective for Molecular Imaging. J Nucl Med.

[7] Schmidkonz C, Kuwert T, Götz TI (2025). Recent advances in nuclear medicine and their role in inflammatory arthritis: focus on the emerging role of FAPI PET/CT. Skeletal Radiol.

[8] Luo Y, Pan Q, Zhou Z (2023). ^68^Ga-FAPI PET/CT for Rheumatoid Arthritis: A Prospective Study. Radiology.

[9] Atzinger A, Tascilar K, Kleyer A (2025). Synovial fibroblast activation occurs before the onset of rheumatoid arthritis and influences the risk of developing disease. RMD Open.

[10] Kastrati K, Nakuz TS, Kulterer OC (2024). FAPi PET/CT for assessment and visualisation of active myositis-related interstitial lung disease: a prospective observational pilot study. *eClinicalMedicine*.

[11] Corte G, Atzinger A, Temiz SA (2024). Anatomical pattern of entheseal and synovial fibroblast activation in patients with psoriasis and its risk of developing psoriatic arthritis. RMD Open.

[12] Corte G, Atzinger A, Tascilar K (2025). OP0174 Synovial and enthesial fibroblast activation detected by 68Ga-FAPI-04-PET/CT predicts the development of psoriatic arthritis in patients with psoriasis and arthralgia. Ann Rheum Dis.

[13] Taylor W, Gladman D, Helliwell P (2006). Classification criteria for psoriatic arthritis: development of new criteria from a large international study. Arthritis Rheum.

[14] Zabotti A, Fagni F, Gossec L (2024). Risk of developing psoriatic arthritis in psoriasis cohorts with arthralgia: exploring the subclinical psoriatic arthritis stage. RMD Open.

[15] Möller I, Janta I, Backhaus M (2017). The 2017 EULAR standardised procedures for ultrasound imaging in rheumatology. Ann Rheum Dis.

[16] Bruyn GA, Iagnocco A, Naredo E (2019). OMERACT Definitions for Ultrasonographic Pathologies and Elementary Lesions of Rheumatic Disorders 15 Years On. J Rheumatol.

[17] Di Matteo A, Di Donato S, Smerilli G (2025). Relationship Between Ultrasound and Physical Examination in the Assessment of Enthesitis in Patients With Spondyloarthritis: Results From the DEUS Multicenter Study. *Arthritis Rheumatol*.

[18] Schmidkonz C, Rauber S, Atzinger A (2020). Disentangling inflammatory from fibrotic disease activity by fibroblast activation protein imaging. Ann Rheum Dis.

[19] Mizoguchi F, Slowikowski K, Wei K (2018). Functionally distinct disease-associated fibroblast subsets in rheumatoid arthritis. Nat Commun.

[20] Rauber S, Mohammadian H, Schmidkonz C (2024). CD200^+^ fibroblasts form a pro-resolving mesenchymal network in arthritis. Nat Immunol.

[21] Cambré I, Gaublomme D, Schryvers N (2019). Running promotes chronicity of arthritis by local modulation of complement activators and impairing T regulatory feedback loops. Ann Rheum Dis.

[22] Schett G, Rahman P, Ritchlin C (2022). Psoriatic arthritis from a mechanistic perspective. Nat Rev Rheumatol.

[23] McGonagle D, Lories RJU, Tan AL (2007). The concept of a “synovio-entheseal complex” and its implications for understanding joint inflammation and damage in psoriatic arthritis and beyond. Arthritis Rheum.

[24] Ishchenko A, Van Mechelen M, Storms L (2025). Low apolipoprotein A1 and high apolipoprotein B levels indicate specific lipid changes in treatment naïve early psoriatic arthritis. RMD Open.

