# Supplementary material for: Increased synovial and entheseal fibroblast activation detected by 68Ga-FAPI-PET/CT is associated with the development of psoriatic arthritis in psoriasis patients with arthralgia
Source: RMD Open. 2026 Mar 23;12(1):e006567. doi: 10.1136/rmdopen-2025-006567 (PMC13034247; doi:10.1136/rmdopen-2025-006567)

Supplementary materials to accompany:

Increased synovial and entheseal fibroblast activation detected by 68Ga-FAPI-PET/CT is associated with development of Psoriatic Arthritis in Psoriasis Patients with arthralgia

Giulia Corte, M.D.[1,2], Armin Atzinger, M.D.[3], Rita Noversa de Sousa, M.D.[1,2,4], Melek Yalcin Mutlu, M.D.[1,2], Alp Temiz, MSc, M.D.[1,2], Sara Bayat, M.D.[1,2], Maria Gabriella Raimondo, M.D.[1,2], Andreas Ramming, M.D.[1,2], Michael Sticherling, M.D.[2,5], Christian Schmidkonz, M.D.[3,6], Torsten Kuwert, M.D.3, Georg Schett, M.D.[1,2], Koray Tascilar [1,2], Filippo Fagni, M.D.[1,2]

[1] Department of Internal Medicine 3, Friedrich-Alexander University (FAU) Erlangen-Nuremberg and Universitätsklinikum Erlangen, Erlangen, Germany.

[2] Deutsches Zentrum fuer Immuntherapie (DZI), FAU Erlangen-Nuremberg and Universitätsklinikum Erlangen, Germany.

[3] Department of Nuclear Medicine, Friedrich-Alexander University (FAU) Erlangen-Nuremberg and Universitätsklinikum Erlangen, Erlangen, Germany.

[4] Serviço de Medicina Interna, Hospital Pedro Hispano, Unidade Local de Saúde de Matosinhos, Matosinhos, Portugal

[5] Department of Dermatology, Friedrich-Alexander University (FAU) Erlangen-Nuremberg and Universitätsklinikum Erlangen, Erlangen, Germany.

[6] Institute for Medical Engineering, Technical University of Applied Sciences Amberg–Weiden, Weiden, Germany.

Correspondence to:

Giulia Corte, MD, Department of Internal Medicine 3 - Rheumatology and Immunology, Friedrich-Alexander University (FAU) Erlangen-Nürnberg and Universitätsklinikum Erlangen, 91054, Erlangen, Germany. E-mail: giulia.corte@uk-erlangen.de

Supplementary tables and figures

Supplementary Table 1– Study inclusion and exclusion criteria

**Inclusion criteria**
Age ≥ 18 years
Dermatologist- or biopsy-confirmed diagnosis of Psoriasis
Presence of arthralgia at baseline which had been present for at least 3 months
Written informed consent
At least 6 months of clinical follow-up available after ^{68}Ga-FAPI-PET/CT
**Exclusion criteria**
Presence of clinical synovitis
Fulfilment of the CASPAR classification criteria at any point
Introduction or change of cs/b/tsDMARD therapy within the 6 months prior to inclusion
Positive rheumatoid factor and/or anti-citrullinated protein antibodies
Overlapping diagnosis of immune-mediated inflammatory diseases (e.g. rheumatoid arthritis, inflammatory connective tissue diseases, autoinflammatory diseases, vasculitis, crystal arthropathies)
Overlapping diagnosis of Fibromyalgia
Presence of arthralgia exclusively in joints with documented osteoarthritis
Pregnancy

Supplementary Table 2– Prevalence and distribution of FAPI uptake

Sites	Overall		without osteoarthritis sites	
	**N of patients**	**Mean SUV (SD)**	**N of patients**	**Mean SUV (SD)**
Great trochanter	15	3.9 (1.1)	12	4.0 (1.0)
Shoulder	14	4.9 (2.4)	10	4.7 (2.4)
Ankle	13	3.5 (1.5)	8	3.8 (1.6)
Knee	13	5.0 (2.1)	7	5.2 (2.2)
Acromioclavicular joint	12	3.9 (1.8)	6	3.9 (1.6)
L5 vertrebra spinosous process	10	4.7 (2.9)	5	3.9 (1.8)
Metatarsal joints	10	3.3 (1.1)	7	3.3 (1.0)
Tarsal joints	10	3.5 (0.8)	6	3.5 (0.8)
Wrist	10	3.9 (1.9)	10	3.9 (1.9)
Plantar fascia	9	3.1 (0.7)	9	3.1 (0.7)
Elbow	8	3.9 (1.3)	7	3.8 (1.4)
Distal phalangeal joints (hands)	7	3.7 (1.0)	5	3.5 (1.2)
Medial femur condyle	6	3.3 (0.7)	4	3.3 (0.6)
Sternoclavicular joint	6	4.9 (1.9)	4	4.8 (2.1)
Achilles´ tendon insertion	5	3.1 (1.2)	4	2.7 (1.1)
Hip	5	3.5 (0.6)	3	3.1 (0.2)
Long biceps tendon insertion	5	3.3 (1.2)	5	3.3 (1.2)
Proximal phalangeal joints (hand)	5	2.7 (0.6)	5	2.7 (0.6)
Iliosacral joint	4	3.7 (1.1)	3	3.8 (1.3)
Metacarpophalangeal joints	4	2.9 (0.6)	3	3.2 (0.3)
Proximal phalangeal joints (feet)	4	4.0 (1.8)	4	4.0 (1.8)
Finger extensor tendons	3	3.2 (1.3)	3	3.2 (1.3)
Finger flexor tendons	3	4.2 (0.4)	3	4.2 (0.4)
Lumbar spine	3	2.3 (0.6)	3	2.3 (0.6)
Anterior superior iliac spine	2	3.7 (0.4)	2	3.7 (0.4)
Lateral epicondyle (elbow)	2	3.2 (0.1)	2	3.2 (0.1)
Patellar tendon insertion	2	7.8 (3.8)	1	11.9 (NA)
Quadriceps tendon insertion	2	3.5 (0.8)	2	3.5 (0.8)
Medial epicondyle (elbow)	1	2.6 (NA)	1	2.6 (NA)
Fibularis tendons	1	2.6 (NA)	1	2.6 (NA)
Posterior superior iliac spine	0	NA	0	NA
Thoracic spine	0	NA	0	NA
Cervical spine	0	NA	0	NA
Temporomandibular joint	0	NA	0	NA
Sternocostal joints	0	NA	0	NA
Iliac crest	0	NA	0	NA

Supplementary Table 3– Prevalence and distribution of ultrasound changes

Ultrasound change	n (%)
Ultrasound Synovitis	24/385 (6,2)
Synovial Power-Doppler	8/385 (2,1)
Ultrasound Tenosynovitis	3/124 (2,4)
Tenosynovial Power-Doppler	0/124 (0)
Entheseal Power-Doppler	7/256 (2,7)
Entheseal Hypoechogenicity	32/256 (12,5)
Entheseal Thickening	12/256 (4,7)
Entheseal Calcifications/Enthesophytes	27/256 (10,5)
Entheseal Erosions	4/256 (1,6)
**Site**	**n**
**Wrist**	
Synovitis	18
Power-Doppler	6
**MCPs**	
Synovitis	6
Power-Doppler	2
**Elbow**	
Synovitis	0
Porwer-Doppler	0
**Knee**	
Synovitis	0
Porwer-Doppler	0
**Finger flexor tendons**	
Greyscale Tenosynovitis	3
Power-Doppler	0
**Epicondylus Humeri lateralis**	
Power-Doppler	3
Hypoechogenicity	13
Thickening	6
Calcification/Enthesophytes	13
Erosions	0
**Quadriceps Tendon insertion on proximal patella**	
Power-Doppler	0
Hypoechogenicity	6
Thickening	2
Calcification/Enthesophytes	3
Erosions	0
**Patellar tendon insertion on distal patella**	
Power-Doppler	0
Hypoechogenicity	3
Thickening	3
Calcification/Enthesophytes	1
Erosions	1
**Achilles´ Tendon enthesis**	
Power-Doppler	4
Hypoechogenicity	10
Thickening	1
Calcification/Enthesophytes	10
Erosions	2

Supplementary Table 4 - Cumulative SUVmax burden in patients who have vs have not developed PsA

**Characteristic**	**Overall** N = 45	**No PsA** N = 26	**PsA** N = 19	**Difference**[2]	**95% CI**[2]	**p-value**[3]
**including ^{68}Ga-FAPI-04 uptake in OA joints**						
Total SUV burden	22 (24)	19 (22)	26 (26)	-0.26	-0.86, 0.34	0.2
Axial SUV burden	0.50 (1.22)	0.54 (1.33)	0.44 (1.11)	0.08	-0.52, 0.68	>0.9
Joint SUV burden	14 (16)	14 (16)	15 (17)	-0.10	-0.70, 0.49	0.6
Entheseal SUV burden	7 (11)	5 (7)	10 (14)	-0.40	-1.0, 0.20	0.4
**excluding ^{68}Ga-FAPI-04 uptake in OA joints**						
Total SUV burden	15 (21)	10 (16)	22 (24)	-0.62	-1.2, -0.01	0.004
Axial SUV burden	0.26 (1.01)	0.20 (1.00)	0.34 (1.04)	-0.14	-0.73, 0.46	0.4
Joint SUV burden	10 (13)	6 (9)	14 (17)	-0.57	-1.2, 0.03	0.047
Entheseal SUV burden	5.4 (9.2)	3.3 (6.5)	8.0 (11.5)	-0.52	-1.1, 0.09	0.14

[1]Mean (SD)

[2]Standardized Mean Difference

[3]Wilcoxon rank sum test

Abbreviation: SUV = Standardized Uptake Value; CI = Confidence Interval

Supplementary Figure 1 - Kaplan-Meier survival estimates for Psoriatic Arthritis development by presence of ultrasound changes. The red line represents patients who presented no signs of pathology at musculoskeletal ultrasound at baseline ("US negative"), while the blue line represents patients presenting any abnormalities at musculoskeletal ultrasound at baseline ("US positive"). The y-axis shows the probability of PsA-free survival. The x-axis denotes follow-up time in months.

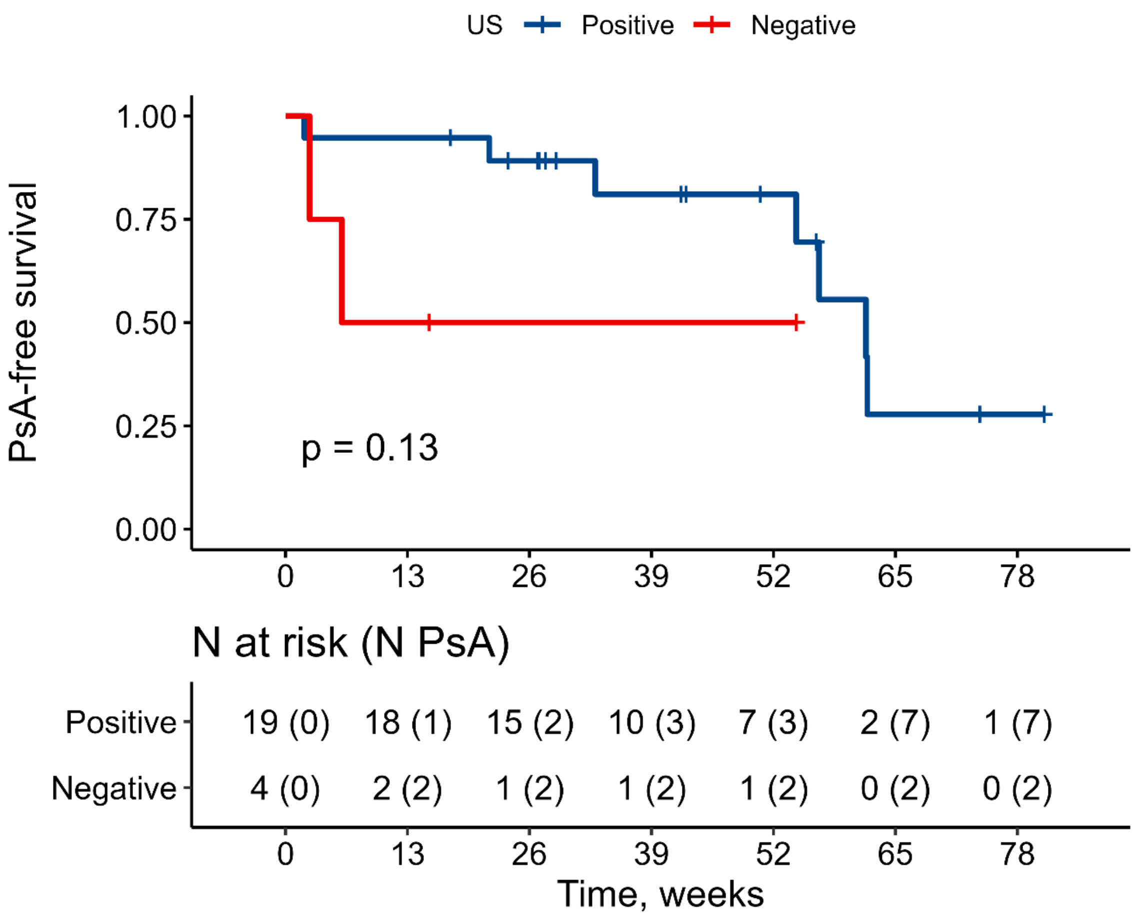

Supplement: online supplemental material 1 [file rmdopen-12-1-s001.pdf]
